# Mental distress and associated factors among Aksum University students, Ethiopia: a cross-sectional study

**DOI:** 10.1186/s12888-019-2051-5

**Published:** 2019-02-13

**Authors:** Tadis Brhane Tesfahunegn, Etsay Hailu Gebremariam

**Affiliations:** 1 Department of Public Health, Aksum University College of Health Science and Referral Hospital, P.O. Box: 1010, Aksum, Ethiopia; 2 Department of Psychiatric Nursing, Aksum University College of Health Science, Referral Hospital, P.O. Box: 1010, Aksum, Ethiopia

**Keywords:** Mental distress, students, prevalence, Ethiopia

## Abstract

**Background:**

Mental distress is becoming a common health problem in Ethiopia. The prevalence of mental distress is higher among university students than the general population. However, there is inadequate information in this regard in Ethiopia situations. The aim of this study was to determine the prevalence and associated factors of mental distress among regular undergraduate students in Aksum University, North Ethiopia.

**Methods:**

An institution based cross-sectional study was carried out among 919 students from May 10 to 20, 2016. Students were selected by stratified multi-stage sampling technique. Data were collected using pre-tested and structured self-administered questionnaire. The collected data were entered into EPI-INFO version 7 and then exported to SPSS version 21 for analysis. Bivariate and multivariate binary logistic regression models were used to determine the factors associated with mental distress among university students.

**Results:**

The prevalence of mental distress was nearly four in ten (39.6%) students. Being female sex (AOR =1.7, 95% CI: 1.20, 2.34), low social support (AOR = 3.05, 95% CI: 1.97, 4.72), freshman students (AOR = 1.73, 95% CI: 1.10, 2.71), having had conflict with friends (AOR = 1.5,95% CI: 1.03, 1.90), financial problem (AOR = 2.20, 95% CI: 1.59, 2.92), family history of mental disorder (AOR = 2.10, 95% CI: 1.37, 3.21), scoring lower grade (AOR = 1.51,95% CI: 1.03, 1.61), argument with instructors (AOR = 1.52,95% CI: 1.12, 2.07) and field of study (AOR = 1.56, 95% CI: 1.03, 2.37) were significantly associated with mental distress.

**Conclusion:**

This study has shown that the magnitude of mental distress among Aksum University students was high. Several social and economic factors were significantly associated with mental distress of the students. Therefore, mental distress needs special focus and timely corrective action by policy makers, university officials, and other concerned stakeholders.

## Background

Mental distress is an emotional state which manifests with different levels of depression, anxiety, panic or somatic symptoms such as sleep problems, headache and backache [1–3]. These may have health effects on the level of an individual’s functioning, and affects many aspects of life, including significant interference with their relationships with other people, and their pleasure of life [2, 4, 5].

Current trends show that the burden of mental health problem is significantly growing worldwide. According to the World Health Organization (WHO) report, mental health problems account to about one-third of disability in the world. The life time prevalence of mental distress all over the world has been estimated as 25% [4]. In Africa, mental distress is a major health problem which accounts for 5% of the total burden of disease and 19% of all disability [6]. However, it is under- recognized as a public burden. Studies show that the prevalence of mental distress is higher among university students than the general population. For instance, among the university students greater than 50% in United States of America (USA) [7],50% in Singapore [8],19.2% in Australia [9], 44.7% in Brazil [10], 41.9% in Malaysia [11], 26.9% in Nigeria [12], 19.8% in Somaliland [13] and10.8% in Kenya[14]. In Ethiopia, the prevalence of mental distress among students was found to be 22 to 49% [15–18].

In Ethiopia there are only two studies on mental distress conducted among under graduate medical students [17, 18]. Few studies carried out in Ethiopia in order to estimate the prevalence of mental distress in the community settings using Self-Reporting Questionnaire (SRQ-20) reported that the prevalence of mental disorders was 17.7 and 25.8% [19, 20].

Various academic, economic, social and psychological factors could be associated with mental distress among university students. These factors could include unfavorable student and teacher relationships, intolerable course load, academic performance of the students, fear of failure, economic problems, problems of adjustment with the environment and conflict with friends [15, 16, 19]. A variety of other factors such as sex, level of education (year of enrollment), and family history of mental disorder could also be related with mental illness [16, 20]. However, little is known about the magnitude and the factors associated with mental distress among university students in Ethiopia [16, 21]. Therefore, the aim of this study was to determine the prevalence and associated factors of mental distress among students in Axum University, Ethiopia.

## Methods

An institution based cross-sectional study was carried out among students to determine the prevalence of mental distress and associated factors from May 10–20, 2016 in Aksum University, North Ethiopia. Aksum University is found at the northern ancient town of Aksum. Aksum town is located at about 1010 km North of Addis Ababa, the Capital city of Ethiopia. In the 2015/2016 academic year, the university had enrolled a total of 10,495 regular undergraduate students under 51 departments. The source population of the study was all regular undergraduate students registered for 2016 academic year in Aksum University. Thus, the study population was all undergraduate regular students registered for 2016 academic year drawn from the selected 22 departments. All regular students who attend the class were included in the study, but students who absent during study period were excluded.

The sample size was determined by using single population proportion formula with the assumptions: 40.9% prevalence of mental distress [16], 95% confidence level,4.5% margin of error, design effect of two and 10% non-response rate. Accordingly, the final sample size of 966 students was used. The study subjects were selected using multi-stage sampling technique. During the first stage, 22 departments were selected from the 51 departments by using simple random sampling. In the second stage, departments in each field of study were further stratified by their years of study. The total sample size was distributed proportionally to each of the departments. Finally, by taking students’ registration number as sampling frame, students were randomly selected. Data were collected using structured self-administered questionnaire. The first part was socio-demographic characteristics. The second part was a Self-Reporting Questionnaire (SRQ-20). SRQ-20 was questionnaire originally developed by the World Health Organization for the screening of mental distress [22]. It consists of 20 item questions (headaches, lack of appetite, sleep problem, being frightened, shaking hands, feel nervous, poor digestion, not thinking clearly, feel unhappy, cry more than usual, difficult to enjoy daily activities, difficulty with decision making, daily work suffering, not feeling life is useful, feeling worthless person, thinking of ending life, loss of interest in life, always feeling tired, uncomfortable feelings in the stomach, and easily tiring) [22]. Therefore, in this section study subjects were asked whether they experience each of the above-mentioned symptoms or not in the last *30- day recall period*. Finally, a total score was computed and the cut-off point for the tool was taken students who had as the presence 8 or more symptoms were considered as having mental distress [16, 17, 23–25]. The questionnaire was validated in developing countries including Ethiopia. The sensitivity of the tool ranges (79–85.7%) and specificity from (66–96%) was found to be acceptable [23–25].

The third part of the questionnaire was about academic and behavioral related factors which included a history of substance use, overloaded with class room lessons, scoring low grade than anticipated, missed too many class or lectures, anticipation of graduation, serious arguments with instructors, and cumulative grade [3, 16, 19]. The last part of the questionnaire was regarding the social support questions assessed by 12 items each having 5 dimensional scale [26]. The reliability of the tool was checked by using Cronbach’s alpha reliability test, and the score was found to be 0.884 (95% CI 0.873–0.895).

Data were collected by six nurse professionals who were trained thoroughly on the data collection procedures. Two masters’ psychiatric nursing instructors together with the principal investigator closely supervised the overall data collection process. The questionnaire was pre-tested, prior to the actual data collection time on 50 undergraduate students that were not included in the main survey. Based on the feedback obtained from the pre-test, appropriate modification was made on the questionnaire.

Data exploration and cleaning was done before analysis. The collected data were checked, coded and entered into EPI INFO version 7, and then exported to SPSS version 21 for analysis. Descriptive statistics like percentage, mean and standard deviation were computed to summarize the data. Multivariate binary logistic regression model was fitted to control the possible effect of confounders for those variables with *P*-value less than or equal to 0.2 during binary logistic regression analysis. Variables with a *P* value less than 0.05 were declared to have a statistically significant association. The model fitness was checked using Hosmer and Lemeshow goodness of fit test statistic (*P* = 0.77).

## Results

### Socio-demographic and related characteristics of respondents

Out of the 966 students (47 participants were excluded due to incomplete response), 919 completed the questionnaires properly which resulted in a total response rate of 95.1%. Of all the respondents 597 (65%) were males. The mean age (±SD) of the students was 21.5(+ 1.91) years. The majority of the students (60.2%) were from urban. More than eight in ten of the students, (85.7%) were Orthodox Christians by religion. Four hundred seventy-three (51.5%) of the students were from the college of Engineering Technology, and two-hundred and seventy six (30%) were freshman students. Majority of the students 774 (84.2%) joined to the different department by, their choices. One hundred and twenty-eight (13.9%) of the students had a family history of mental illness and 421 (62.3%) of the students received some monthly pocket money from their family (Table 1).

**Table 1:** Socio-demographic characteristics of students, Aksum University, North Ethiopia, May 2016 (*n* = 919)

Variables	Categories	Frequency	Percentage
Age in year	≥20 21–24 ≥25	116 738 65	12.6 80.3 7.1
Sex	Male Female	597 322	65.0 35.0
Residence	Urban Rural	559 360	60.2 39.8
Religion	Orthodox Christian Muslim Protestant Other	785 58 66 10	85.4 6.3 7.2 1.0
College/faculty/school	Medicine and health sciences Business and economics Natural & computational sciences Social sciences and humanities Agriculture Engineering	94 134 77 85 56 473	10.2 14.6 8.4 9.2 6.1 51.5
Department choice	Preferred Not preferred	774 145	84.2 15.2
Year of enrollment	1st 2nd 3rd 4th	274 228 177 240	29.8 24.8 19.3 26.1
Interest of the department	Yes No	780 139	84.9 15.1
Religion practice	Yes No	484 435	52.7 47.3
Conflict in the dormitory	Yes No	443 476	48.2 51.8
Have pocket money	Yes No	383 536	41.7 58.3
Financial distress	Yes No	443 476	48.2 51.8
Family history of mental illness	Yes No	128 791	13.9 86.1

### Prevalence of mental distress and students’ academic performance

The prevalence of mental distress among the university students was 364 (39.6% with (95% CI: 36.6–42.9). Two hundred-sixty three (28.6%), 234 (25.5%) and 422 (45.9%) students reported low, moderate and high social support respectively (Table 2).

**Table 2:** Prevalence of mental distress, social support, and student’s performance among Aksum University students, Northern Ethiopia May 2016 (n = 919)

Variable	Categories	Frequency	Percentage
Mental distress			
	Yes No	364 555	39.6 60.4
Social support			
	High level Moderate level Low level	234 422 263	25.5 45.9 28.6
Student’s performance	< 2.95 > 2.95	435 484	47.3 52.7

### Academic related factors

Above one third 314 (34.2%) of the respondents reported that they were overloaded with classroom lessons during the study period. Four hundred thirty-four (47.2%) of the respondents reported they had lower grades than they expected. Likewise, 505 (55%) of the respondents were reported that they had conflicts with their instructors (Table 3).

**Table 3:** Academic related factors of respondents, Aksum University, Northern Ethiopia May 2016(n = 919)

Variable	Categories	Frequency	Percentage
Overloaded with class room lessons			
	Yes No	314 605	34.2 65.8
Low grade than anticipated			
	Yes No	434 485	47.2 52.8
Miss too many class			
	Yes No	203 716	22.1 77.9
Anticipation of graduation			
	Yes No	680 239	74.0 26.0
Have strained relationship with instructors			
	Yes No	505 411	55.0 45.0
Had vacation			
	Yes No	505 414	55.0 45.0

### Factors associated with mental distress

The multivariate logistic regression analysis showed that being female students (AOR = 1.70, 95% CI: 1.2, 2.34), being a student in college of agriculture (AOR = 0.37, 95% CI: 0.17, 0.83), being freshman (first year) students (AOR = 1.73, 95% CI: 1.10, 2.71), interest towards the department (AOR = 1.56, 95% CI: 1.03, 2.37), conflict with friends (AOR = 1.50, 95% CI: 1.03, 1.90), having financial problems (AOR = 2.20, 95% CI: 1.59, 2.92), having family history of mental disorder (AOR = 2.10,95% CI: 1.37, 3.21), scoring lower grade than anticipated (AOR = 1.51, 95% CI: 1.03, 1.61), no social support (AOR = 3.05, 95% CI: 1.97, 4.72) and argument with instructors (AOR = 1.52, 95% CI: 1.12, 2.07) were found significantly linked with mental distress (Table 4).

**Table 4:** Bivariate and multivariate logistic regression analysis of factors associated with mental distress among students, Aksum University, Northern Ethiopia, May 2016 (n = 919)

Variable	Categories	Mental distress Yes No		COR (CI: 95%) Crude	AOR (CI: 95%) Adjusted
Sex					
	Male Female	227 137	370 185	1 1.21(0.92, 1.59)	1 1.7(1.20, 2.34)*
College/faculty/					
	Health Business Natural Social sciences Agriculture Engineering	34 56 36 43 11 184	60 78 41 42 45 289	0.89 (0.56, 1.41) 1.13(0.76, 1.67) 1.38 (0.85, 2.24) 1.61(1.01, 2.57) 0.34 (0.19, 0.76)* 1	0.97 (0.56, 1.69) 1.27 (0.75, 2.18) 1.22 (0.66, 2.28) 1.39 (0.76, 2.57) 0.37(0.17, 0.83) * 1
Year of enrolment					
	1st 2nd 3rd 4th	121 94 64 85	153 134 113 155	1.44 (1.01, 2.06)* 1.28 (0.88, 1.86) 1.03 (0.69, 1.55) 1	1.73 (1.10, 2.71)* 1.36 (0.85, 2.17) 1.21 (0.73, 1.20) 1
Interested with the department					
	Yes No	291 73	489 66	1 1.86 (1.29, 2.67)*	1 1.56 (1.03, 2.37)*
Financial distress/problem/					
	Yes No	224 140	219 336	2.46 (1.87, 3.22)* 1	2.20 (1.59, 2.92)* 1
Religious practice					
	Yes No	175 246	309 189	1 1.36 (1.04, 1.77)*	1 1.10 (0.81, 1.51)
Ever had conflict with friends					
	Yes No	198	245	1.51 (1.16, 1.97)* 1	1.5 (1.03, 1.90) * 1
Family history of mental illness					
	Yes No	77 287	51 504	2.65 (1.81, 3.89)* 1	2.10 (1.37, 3.21)* 1
Social support					
	High level Moderate level Low level	55 156 153	179 266 110	1 1.91(1.33, 2.74)* 4.53(3.07, 6.68)*	1 1.65 (1.17, 2.43)* 3.05 (1.97, 4.72)*
Life time khat use					
	Yes No	57 307	60 495	1.53(1.04, 2.61)* 1	1.30 (0.77, 2.12) 1
Life time alcohol use					
	Yes No	283 181	242 313	1.31 (1.03, 1.71)* 1	1.01 (0.64, 1.50) 1
Overloaded with class room lessons					
	Yes No	145 219	169 386	1.51(1.15, 1.99)* 1	1.30 (0.91, 1.86) 1
Lower grade than anticipated					
	Yes No	188 176	246 309	1.34 (1.10, 1.75)* 1	1.51 (1.03, 1.61)* 1
Miss too many class					
	Yes No	105 259	98 457	1.89(1.38–2.59)* 1	1.29(0.89–1.88)) 1
Argument with instructors					
	Yes No	168 196	176 379	1.85(1.41–2.43)* 1.00	1.52(1.12–2.07)*

*Remained significance when adjusted for other variables in the table

## Discussion

In this study, the prevalence of mental distress at Aksum University was nearly four in ten (39.6%) students. This is relatively lower than that of previous other studies in India 53% [27], and Brazil 44.7% [10]. This variation might be due to the difference of mental health policy, socio-cultural, environmental and economic background or the differences in the use of the cutoff point of SRQ − 20 tools. The level of mental distress reported by this study was lower than the finding from previous study done among local university students in Hawassa, Ethiopia, which reported the prevalence of mental distress was 49% [15]. This difference in magnitude might be due to the time difference between the studies. The study in Hawassa, Ethiopia was conducted ten years before this study. On the contrary, this finding was higher than the prevalence of mental distress which was reported among undergraduate students in French university 25.7% [20], Norwayan University 22.9% [28], Australian University 19.2% [9], Somaliland University 19.8% [13], Nigerian University 26.9% [12] and Ethiopia University 21.6% [3]. The discrepancy observed could be due to cut-off point of SRQ − 20 or tool difference used in the different studies.

However, this finding is comparable with similar study conducted among university students in Ethiopia, 40.9% [16] which used a similar tool and cut-off point. Also, a study from Malaysia had reported a similar prevalence of emotional disorders of 41.9% [11].

In this study, sex of the students found to be significantly associated with mental distress.

Being female student is 1.7 times more likely to develop mental distress than being male student. The finding of this study in concurrence with similar studies done on university students in Australia [9], France [20], Norway [28] and Egypt [29] which showed that female students had higher levels of mental distress than male students. The possible reasons for these sex differences might be the biological nature of their responses to stressors and environmental risk factors such as violence [30].

In this study, year of study was one of the factors that influenced mental health condition. Being a freshman (first year) student was 1.7 times more likely to develop mental distress as compared to final year university students. Study findings in this areas showed that being freshman student was associated with higher levels of psychological distress than being senior student [17, 31]. This could be explained by the fact that first-year students face new academic challenge, new social relations and separation from earlier family connection when adapting to a new environment in the university [16].

In addition, this study showed that students’ interest towards the field of study was statistically associated with mental distress. University students who were not interested in their field of study were 1.56 times higher to have mental distress than their counterparts. Similar findings were also reported in other parts of Ethiopia [3, 16]. Furthermore, students who reported history of mental illness among family members in the past were two times more likely to develop mental distress than students who report no family history of mental illness. Similar study findings in Ethiopia showed that family history of mental illness was significantly associated with mental distress of students [3, 16]. This might be explained by the genetic causes [32].

In this study, students who had financial problems were two times more likely to develop mental distress as compared with students with good financial status. Other studies reported a similar finding on the association between economic problems and mental distress [15, 16]. This might be due to the fact that raising financial costs of stationary materials and other expenditures as these might have generated a psychological stress on students [15, 33, 34]. On the other hand, earlier conflict with their roommates was associated with mental distress. Those students who had conflict with their roommates’ showed higher levels of mental distress than students who had not any conflict. Previous studies from Ethiopian Universities also reported the same experience on the association of roommates’ conflict and mental distress [3, 16]. This might be due to the fact that, increased peer pressure in campus life where students live together in a groups may in turn lead to emotional stress [16]. Similarly, one important finding in this study revealed that argument with instructors was associated with mental distress. This finding was supported by previous study which showed that conflict between students and instructors negatively affected the students` psychology, social behavior and their academic achievement [35].

This study finding reported that academic factors are significantly associated with mental distress of university students. Students who score grades lower than their anticipation were 1.5 times more likely to have mental distress than those students who score better grades. This was in line with reports from other studies conducted in Ethiopia [3, 16].

This study finding also indicated that social support was significantly associated with mental distress. Students who obtain low social support were three times more likely to have mental distress as compared to those students with high social support [16, 28, 36]. This could be due to social factors that can promote health through stress inhibiting the adverse physiological effects by providing a sense of belonging through communications [37].

### Limitations

Reports on some of the questions were from a past history, which may be prone to recall bias. The other point is that there might be social desirability bias due to sensitive questions related to substance use. The study design used descriptive cross-sectional type, thus, it is difficult to confirm about the causal relationship.

## Conclusion

This study has shown that the prevalence of mental distress among Aksum University students was high. Being female sex, freshman student, field of study, having had conflict with friends, having financial problem, family history of mental disorder, scoring lower grade, argument with instructors and low to moderate social support was associated with mental distress of university students. Therefore, mental distress needs special focus and timely corrective action from policy makers, university officials, and other concerned stakeholders. Interventions targeting to strengthening social and financial support that reduces the prevalence of mental distress of university students are recommended.

## Abbreviations

CI: confidence interval
AOR: adjusted odds ratio
COR: crude odds ratio
CGPA: Cumulative grade point average
SRQ: self-reporting questionnaire
WHO: World health organization
USA: United States of America
